# Protracted Outbreak of *Salmonella* Newport Infections Linked to Ground Beef: Possible Role of Dairy Cows — 21 States, 2016–2017

**DOI:** 10.15585/mmwr.mm6715a2

**Published:** 2018-04-20

**Authors:** Katherine E. Heiman Marshall, Mackenzie Tewell, Selam Tecle, Molly Leeper, Jennifer Sinatra, Bonnie Kissler, Adrienne Fung, Kerri Brown, Darlene Wagner, Eija Trees, Kelley B. Hise, Vishnu Chaturvedi, Linda K. Schlater, Brenda R. Morningstar-Shaw, Laura Whitlock, Kristin Holt, Karen Becker, Megin Nichols, Ian T. Williams, Michael Jhung, Matthew E. Wise, Laura Gieraltowski

**Affiliations:** ^1^Division of Foodborne, Waterborne, and Environmental Diseases, National Center for Emerging and Zoonotic Infectious Diseases, CDC; ^2^Arizona Department of Health Services; ^3^California Department of Public Health; ^4^Food Safety and Inspection Service, U.S. Department of Agriculture; ^5^Texas Department of State Health Services; ^6^Colorado Department of Public Health; ^7^California Department of Public Health Microbial Diseases Laboratory; ^8^National Veterinary Services Laboratory, Animal and Plant Health Inspection Service, U.S. Department of Agriculture.

In January 2017, CDC identified a cluster of *Salmonella enterica* serotype Newport infections with isolates sharing an indistinguishable pulsed-field gel electrophoresis (PFGE) pattern, JJPX01.0010 (pattern 10), through PulseNet, the national molecular subtyping network for foodborne disease surveillance. This report summarizes the investigation by CDC, state and local health and agriculture departments, and the U.S. Department of Agriculture’s Food Safety and Inspection Service (USDA-FSIS) and discusses the possible role of dairy cows as a reservoir for strains of *Salmonella* that persistently cause human illness. This investigation combined epidemiologic and whole genome sequencing (WGS) data to link the outbreak to contaminated ground beef; dairy cows were hypothesized to be the ultimate source of *Salmonella* contamination.

## Epidemiologic Investigation

**FIGURE 1:**
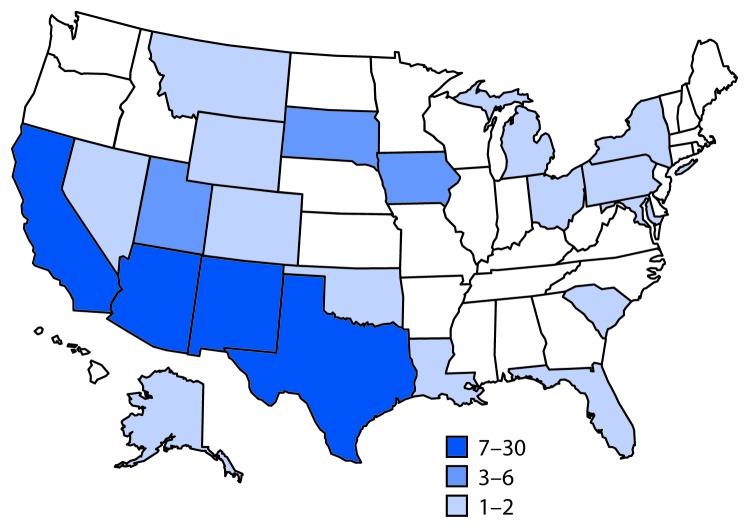
Infections with the outbreak strain of *Salmonella* Newport (n = 106), by state of residence — 21 states, October 2016–July 2017

**FIGURE 2:**
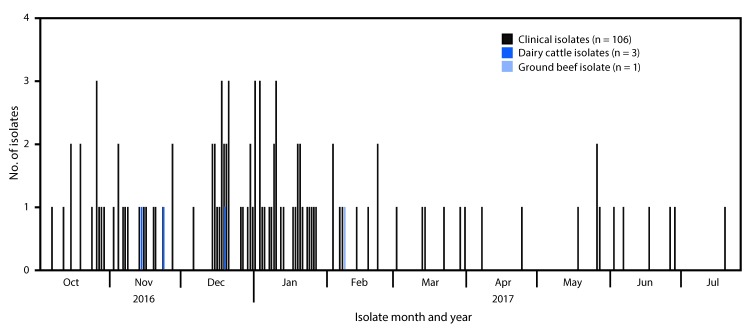
Isolates of the outbreak strain of *Salmonella* Newport from patients (n = 106), dairy cattle* (n = 3), and leftover ground beef (n = 1) — 21 states, October 2016–July 2017 *The isolate collected from a dairy cow fetus in July 2016 is not displayed because cases were reported during July–October 2016 but were not investigated as part of this outbreak.

A case was defined as infection with *Salmonella* Newport with PFGE pattern 10 closely related to the outbreak strain by WGS, with bacterial isolation during October 1, 2016, through July 31, 2017. A total of 106 cases were identified in 21 states (Figure 1). Most illnesses ([72%]) were reported from southwestern states, including Arizona (30), California (25), New Mexico (14), and Texas (seven). Illness onset dates ranged from October 4, 2016, through July 19, 2017 (Figure 2). Patients ranged in age from <1–88 years (median = 44 years), and 53 (50%) were female. Among 88 (83%) patients with known outcomes, 42 (48%) were hospitalized, and one died.

Initial interviews identified consumption of ground beef as a common exposure among patients. A focused questionnaire was developed to collect detailed information on ground beef exposure and to obtain shopper card information and receipts. Among 65 interviewed patients, 52 (80%) reported eating ground beef at home in the week before illness began. This percentage was significantly higher than the 2006–2007 FoodNet Population Survey, in which 40% of healthy persons reported eating ground beef at home in the week before they were interviewed (p<0.001) (*1*). Among the 52 patients who ate ground beef at home, 31 (60%) reported that they bought it or maybe bought it from multiple locations of two national grocery chains, and 21 (40%) reported that they bought ground beef from locations of 15 other grocery chains. Specific ground beef information was available for 35 patients. Among these, 15 (43%) purchased ground beef as chubs (rolls) of varying sizes (range = 2–10 lbs), 18 purchased it on a tray wrapped in plastic, and two purchased preformed hamburger patties. Twenty-nine patients reported that they bought fresh ground beef, four bought frozen ground beef, and four did not recall whether it was fresh or frozen when purchased. When asked about ground beef preparation, 12 (36%) of 33 patients reported that they definitely or possibly undercooked it.

## Traceback Investigation

USDA-FSIS conducted traceback on ground beef purchased within 3 months of illness onset for 11 patients who provided shopper card records or receipts. Approximately 20 ground beef suppliers belonging to at least 10 corporations were identified; 10 of the 11 records traced back to five company A slaughter/processing establishments, seven of 11 traced back to five company B slaughter/processing establishments, and four of 11 traced back to two company C slaughter/processing establishments.

## Product and Animal Testing

Opened, leftover samples of ground beef from three patients’ homes were collected for testing. All were purchased from one of two national grocery chains that had been identified by a majority of patients. One sample, collected from ground beef removed from its original packaging, yielded the outbreak strain. The other two samples did not yield *Salmonella*.

The outbreak strain was also isolated from four New Mexico dairy cattle (Table). One was collected from a spontaneously aborted fetus in July 2016, and one was isolated from feces from a young calf in November 2016. The third isolate was identified by searching the USDA Animal and Plant Health Inspection Service National Veterinary Services Laboratory (USDA-APHIS NVSL) database for *Salmonella* Newport isolates collected from cattle in Arizona, California, Texas, New Mexico, and Wisconsin during January 2016–March 2017. Eighteen *Salmonella* Newport isolates were identified, including 13 from Texas, three from New Mexico, and two from Wisconsin. The only *Salmonella* Newport pattern 10 isolate identified was from a fecal sample from a New Mexico dairy cow collected during November 2016. The fourth isolate was from a USDA-FSIS routine cattle fecal sample collected at a Texas slaughter establishment in December 2016; USDA-FSIS determined the sample was from a dairy cow and identified the New Mexico farm of origin. Because of confidentiality practices, officials were not able to identify the farm or farms of origin for the dairy cows associated with the other three samples or whether the four dairy cows were associated with a single farm. None of the 11 patients with information for traceback ate ground beef produced at the Texas slaughter establishment.

**TABLE:** *Salmonella* Newport pattern 10 isolates with the outbreak strain collected from dairy cattle sourced from New Mexico, 2016

Isolate no.	Collection site	Isolation date	Sample source or reason for collection
1	Fetal tissue	Jul 7, 2016	Necropsy of cow fetus
2	Feces	Nov 14, 2016	Young calf
3	Feces	Nov 23, 2016	Cattle of unknown age collected because of infection*
4	Cecum	Dec 19, 2016	Routine sampling at slaughter facility in Texas; cow traced to New Mexico

**Abbreviation:** NVSL = USDA Animal and Plant Health Inspection Service National Veterinary Services Laboratory.

*Because of the anonymity of samples from cows routinely tested by NVSL, it is possible that this isolate is from the same sample as isolate 2.

## Laboratory Investigation

Whole genome high-quality single nucleotide polymorphism (SNP) analysis* showed that 106 clinical isolates were closely related to each other genetically, to the four dairy cattle isolates, and to the leftover ground beef isolate (range = 0–12 SNP differences), suggesting that the Salmonella bacteria found in patients, ground beef, and dairy cattle all shared a common source. Thirty-nine additional clinical isolates with PFGE pattern 10 were determined to not be closely related and were excluded from the outbreak. No antibiotic resistance was detected among three clinical isolates tested by CDC’s National Antimicrobial Resistance Monitoring Laboratory.^†^

## Public Health Response

Because the USDA-FSIS traceback investigation did not converge on a common production lot of ground beef or a single slaughter/processing establishment, and no ground beef in the original packaging yielded the outbreak strain, a recall of specific product was not requested. A public warning was not issued to consumers because specific, actionable information was not available (e.g., a specific brand or type of ground beef). Officials in New Mexico visited the dairy farm that was the source of the cow at the Texas establishment and noted no concerns about conditions or practices. However, this visit occurred late in the investigation, and conditions at the time of the visit might not have represented those present immediately before and during the outbreak. No samples from the environment or cows were collected during this visit.

## Discussion

Epidemiologic and laboratory evidence indicated that contaminated ground beef was the likely source of this protracted outbreak of *Salmonella* Newport infections. A significantly higher percentage of patients than expected ate ground beef at home, and a patient’s leftover ground beef yielded the outbreak strain. Dairy cows colonized or infected with the outbreak strain before slaughter are hypothesized to be the ultimate outbreak source. Most U.S. ground beef is produced from beef cattle; however, 18% is produced from dairy cows (*2*). Dairy cows are sold for beef production through sale barns or directly to slaughter establishments as they age or if their milk production is insufficient (*2*). Previous studies have demonstrated long-term persistence of *Salmonella* Newport in dairy herds (*3*,*4*), and a 1987 *Salmonella* Newport outbreak was linked to contaminated ground beef from slaughtered dairy cows (*5*). In the current outbreak, as has been observed in previous outbreaks, ground beef purchases traced back to numerous lots and slaughter/processing establishments (*6*). One possible explanation is that dairy cows carrying a high *Salmonella* load that overwhelmed antimicrobial interventions could have gone to multiple slaughter/processing establishments (*7*), resulting in contamination of multiple brands and lots of ground beef. This might explain the reason for failure to identify a single, specific source of contaminated ground beef.

This investigation identified the outbreak strain only in samples from dairy cattle from New Mexico. All four isolates from dairy cattle samples were closely related genetically by WGS to isolates from patients, providing further evidence of a connection between dairy cattle in New Mexico and the outbreak. The disproportionate geographic distribution of cases in the U.S. Southwest, including New Mexico, also suggests a possible regional outbreak source. Although limited in scope, the query of the USDA-APHIS NVSL data identified the outbreak strain only from one New Mexico dairy cow (isolate 3), and the sample collection date was consistent with the timing of illnesses in this outbreak. The overall prevalence and geographic distribution of the outbreak strain in cattle is not known, and it is possible that cattle in states other than New Mexico might have been infected or colonized with the outbreak strain.

This was a complex and challenging investigation for several reasons. First, the PFGE pattern in the outbreak was not uncommon in PulseNet, making it difficult to distinguish outbreak cases from sporadic illnesses associated with the same *Salmonella* Newport pattern. WGS analysis provided more discriminatory power to refine the outbreak case definition and excluded 39 cases of illness from the outbreak. However, sequencing is not currently performed in real time for *Salmonella*, thereby slowing the process of determining which cases were likely outbreak-associated. In addition, a direct pathway linking outbreak cases to dairy cows infected with the outbreak strain of *Salmonella* Newport could not be established. This is because product traceback did not converge on a single contaminated lot of ground beef, and investigators were unable to ascertain a link between the beef slaughter/processing establishments identified during traceback and the farms with dairy cows that yielded the outbreak strain. Tracing back ground beef purchased by patients to slaughter/processing establishments requires documentation such as receipts or shopper card records, and only 10% of patients had this information available. For this outbreak, tracing back cows at slaughter/processing establishments to the farm from which they originated was problematic because cows were not systematically tracked from farm to slaughter/processing establishments.

Four points along the “farm to fork” continuum provide opportunities to prevent consumers from becoming ill from contaminated ground beef. First, farms can implement good management practices for cattle health, including vaccination, biosecurity (e.g., controlling movement of persons and animals on farms, keeping a closed herd [so that no animals on the farm are purchased, loaned to other farms, or have contact with other animals], planning introduction of new animals and quarantining them, and performing microbiologic testing of animals), and cleaning and disinfection measures to decrease *Salmonella* burden in animals and the environments in which they reside, reducing the likelihood that *Salmonella* will enter beef slaughter/processing establishments (*8*). Second, slaughter/processing establishments are required to maintain Hazard Analysis and Critical Control Points systems to reduce *Salmonella* contamination as well as slaughter and sanitary dressing procedures to prevent carcass contamination (*9*). Third, although *Salmonella* is not considered an adulterant in not-ready-to eat (NRTE) meat products, USDA-FSIS likely will consider the product to be adulterated when NRTE meat products are associated with an outbreak (*9*). Finally, consumers are advised to cook ground beef to 160°F (71°C) as measured by a food thermometer to destroy any bacteria that might be present. Consumers are also advised to wash hands, utensils, and surfaces often; separate and not cross-contaminate foods; and refrigerate foods promptly and properly.

This investigation emphasizes the utility of WGS during outbreak investigations and identifies the need for improvements in traceability from the consumer to the farm. It also highlights the importance of continued evaluation of farm practices to help reduce persistent *Salmonella* contamination on farms, contamination of ground beef, and ultimately human illness.

**Summary**

**What is already known about this topic?**

Previous outbreaks of salmonellosis were linked to contaminated ground beef produced from slaughtered dairy cows.

**What is added by this report?**

Contaminated ground beef was the likely source of a protracted outbreak of 106 *Salmonella* Newport infections, 42 hospitalizations, and one death in 21 states during October 2016–July 2017. While no direct link was found, whole genome sequencing suggests dairy cows were the ultimate outbreak source.

**What are the implications for public health practice?**

Foodborne outbreak investigations could be enhanced by improvements in the traceability of cows from their originating farms or sale barns, through slaughter and processing establishments, to ground beef sold to consumers.

## Footnotes

* https://github.com/lskatz/lyve-SET.

† https://www.cdc.gov/narms/antibiotics-tested.html.
